# 细针吸取细胞学标本检测非小细胞肺癌*EGFR*、*KRAS*突变的探讨

**DOI:** 10.3779/j.issn.1009-3419.2015.04.05

**Published:** 2015-04-20

**Authors:** 智慧 张, 希兰 吴, 建明 应, 峻岭 李, 田 邱, 会芹 郭, 焕 赵, 灵 山, 云 凌

**Affiliations:** 1 100021 北京，北京中国医学科学院肿瘤医院病理科 Department of Pathology, Tumor hospital Chinese Academy of Medical Sciences, Beijing 100021, China; 2 650031 云南，昆明医科大学病理教研室 Department of Pathology of Medicine University of Kunming, Kunming 650031, China

**Keywords:** 肺肿瘤, 表皮生长因子受体, 细针吸取细胞学, 基因, Lung neoplasms, Epidermal growth factor receptor, Fine needle aspiration cytology, Gene

## Abstract

**背景与目的:**

肺癌患者靶向药物治疗前，需要检测表皮生长因子受体（epidermal growth factor receptor, *EGFR*）和*KRAS*基因是否突变。本研究旨在探讨细针吸取悬浮液标本检测非小细胞肺癌*EGFR*、*KRAS*基因突变的意义。

**方法:**

细胞学悬浮液标本，Real-time PCR法检测EGFR 18-21号外显子，KRAS 2号外显子12、13密码子突变。

**结果:**

检测85例肺癌转移淋巴结针吸标本，*EGFR*突变率37.3%，*KRAS*突变率7.2%。19例组织切片标本，与细胞学结果一致（kappa=1.0）。13例有*EGFR*突变，临床分期Ⅳ期患者，使用酪氨酸激酶抑制剂治疗，2例完全缓解（complete remission, CR）（16.7%）；8例部分缓解（partial remission, PR）（66.7%）；3例最佳稳定疾病（stable disease, SD）（25.0%）。

**结论:**

细针吸取标本检测*EGFR*、*KRAS*基因突变，标本取材容易、简单、方便，具有较高的临床实用性。

## Abstract (en)

**Background and objective:**

Epidermal growth factor receptor (EGFR) and KRAS must be detected mutation status before patients of lung cancer use targeted drugs. The aim of this study is to elucidate the significance of *EGFR* and *KRAS* mutation in fine needle aspiration (FNA) cytology suspension specimens of non-small cell lung carcinoma.

**Methods:**

*EGFR* gene exons 18-21 and *KRAS* codons 12, 13 of exons 2 were performed by Real-time PCR methods in fine needle aspiration cytology suspension specimens of lymph nodes.

**Results:**

85 metastasis lymph nodes were detected in fine needle aspiration cytology samples of lung cancer. *EGFR* mutation rate was 37.3%. *KRAS* mutation rate was 7.2%. 19 formalin fixed paraffin-embedded tissue specimens were available and match cytology specimens. Analysis of *EGFR* mutation status in those samples revealed agreement with the results obtained in cytological samples (kappa=1.0). Clinical follow-up was available for 13 who presented with stage Ⅳ disease. Based on the identification of such mutations, these patients received subsequent therapy with a TKI in clinic. We observed two cases complete remission (16.7%) and 8 cases partial remission (66.7%) and three had ongoing stable disease.

**Conclusion:**

Fine-needle aspiration cytology samples were detected *EGFR* and *KRAS* mutation. The method which collects samples was easier, simple and convenient. This method has higher application value in clinical treatment.

肺癌是世界上发病率和病死率最高的肿瘤^[1]^，其中非小细胞肺癌（non-small cell lung cancer, NSCLC）约占80%，70%患者就诊时已处于肺癌晚期。晚期肺癌患者预后差，采用常规化疗其平均生存期小于1年^[2]^。近年来表皮生长因子受体（epidermal growth factor receptor, EGFR）-酪氨酸激酶抑制剂（tyrosine kinase inhibitor, TKI）为NSCLC的治疗带来了希望，但是用药前必须检测*EGFR*、*KRAS*的突变情况，因为EGFR野生型对TKI药物不敏感，所以检测基因的突变非常必要^[3]^。有些晚期肺癌患者的病理诊断是根据细胞学标本做出的，如：转移淋巴结细针吸取、胸腔积液等，部分患者细胞学标本可能是其唯一的标本来源。能够手术的肺癌患者检测*EGFR*、*KRAS*基因突变采用手术后石蜡包埋组织切片标本，但是对于不适宜手术的晚期肺癌患者其*EGFR*、*KRAS*的基因突变检测，细胞学标本可能是其重要来源。本研究探讨细胞学细针吸取的悬浮液标本检测*EGFR*、*KRAS*突变的临床意义。

## 1 材料与方法

### 1.1 临床资料

标本来自中国医学科学院肿瘤医院病理科细胞学室，自2011年3月-2013年2月间，肺癌患者纵隔和体表转移淋巴结细针吸取，细胞学诊断结果为非小细胞癌（包括鳞癌和腺癌），共85例。纵隔转移淋巴结超声内镜引导下的经支气管针吸活检（endobronchial ultrasound-guided transbrochial needle aspiration, EBUS-TBNA）针吸5例，体表转移淋巴结盲穿针吸80例。其中男性45例，女性40例，年龄31岁-75岁（中位年龄54岁）。同时，19例有手术后石蜡包埋病理切片标本作为匹配进行EGFR、KRAS检测，9例为肺癌原发灶标本，10例为肺癌转移淋巴结活检切除标本。13例有临床应用TKI抑制剂的治疗效果记录。85例均由组织病理或者免疫细胞化学结果证实为鳞状细胞癌4例、腺癌81例。

### 1.2 标本的采集

用22 G一次性注射器（针头规格0.7 mm）吸取淋巴结，吸出物制作传统涂片和液基薄层涂片各1张用于细胞学临床诊断，剩余在PreservCyt保存液中的悬浮液标本留作EGFR、KRAS检测，为了保证检测有足够的肿瘤细胞，并且肿瘤细胞量在70%以上，每例在基因检测前先观察液基薄层涂片中肿瘤细胞的数量和比例是否达到标准。

### 1.3 DNA的提取

使用QIAamp^®^ DNA mini试剂盒（Qiagen公司，德国）提取基因组DNA，操作按照使用说明书进行。使用微量紫外分光光度计测定DNA浓度、纯度。

### 1.4 Real-time PCR分析

使用商品化的人*EGFR*、*KRAS*基因突变检测试剂盒（荧光PCR法）检测*EGFR*、*KRAS*基因突变，基因突变检测试剂盒购自北京雅康博生物科技有限公司，操作按照使用说明书进行。

### 1.5 统计学分析

TKI治疗的反应率采用实体瘤评估反应标准进行统计^[4]^。细胞学样本和组织学样本突变状态的一致性，采用Cohen’s k系数进行比较^[5]^.一致性关系分为：0.81-1.00为完全一致；0.61-0.80为大体一致；0.41-0.60为中等一致^[6]^。

## 2 结果

### 2.1 样本的满意率

共检测转移淋巴结细针吸取标本85例，5例由于提取DNA量不足，无法进行检测，为不满意标本，其中包括EBUS-TBNA针吸2例（2/5），占40%，体表转移淋巴结盲穿针吸3例（3/80），占3.8%。3例盲穿针吸病例经过重新吸取，补充细胞量，使提取DNA量充足，能够进行检测，但是EBUS-TBNA针吸2例未能进行补救。至此，能进行检测的有效标本共83例。我们获得了平均31.06 ng/μL的DNA。

### 2.2 *EGFR*突变

检测肺癌转移淋巴结细针吸取标本83例，*EGFR*基因突变31例，突变率为37.3%。1例同时检测到20号和21号外显子上2个突变点，另1例2个突变点同时位于19号和21号外显子上。31例突变中19号、21号、20号和18号的突变率分别为51.5%（17/33）、42.5%（14/33）、3.0%（1/33）和3.0%（1/33）。19号和21号外显子上*EGFR*基因的突变占94.0%。分析各外显子突变类型，主要为第19号外显子的缺失突变和第21号外显子L858R点突变。*EGFR*基因突变检测结果见图 1A和图 1B。31例突变中，2例为鳞状细胞癌（2/4），突变率50%；29例腺癌（29/79），突变率36.7%。女性突变占女性患者的60.0%（24/40），男性突变占男性患者的16.3%（7/43）。*EGFR*的突变情况及突变率见表 1和表 2。

**1:**
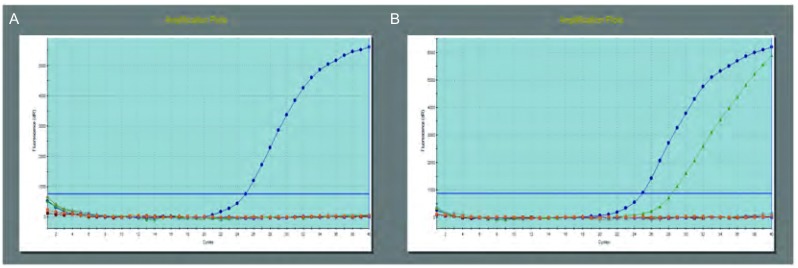
Real-time PCR检测*EGFR*基因突变。A：*EGFR*基因野生型；B：*EGFR*基因突变型。 Real-time PCR detect *EGFR* gene mutation. A: *EGFR* gene wild type; B: *EGFR* gene mutation type

**1:** *EGFR*的突变情况 *EGFR* mutation status

Mutation No.	*EGFR* mutation	Exon	Gender	Age	Sample source	Diag.	DNA source
1	Point	18	F	72	FNA	Adenocarcinoma	Suspension
2	Delete	19	M	72	FNA	Adenocarcinoma	Suspension
3	Delete	19	F	71	FNA	Adenocarcinoma	Suspension
4	Insert	19	F	55	FNA	Adenocarcinoma	Suspension
5	Delete	19	F	62	FNA	Squamous cancer	Suspension
6	Delete	19	F	51	FNA	Adenocarcinoma	Suspension
7	Delete	19	F	75	FNA	Adenocarcinoma	Suspension
8	Delete	19	F	52	FNA	Adenocarcinoma	Suspension
9	Delete	19	F	49	FNA	Adenocarcinoma	Suspension
10	Delete	19	F	50	FNA	Adenocarcinoma	Suspension
11	Point	19	F	50	FNA	Adenocarcinoma	Suspension
12	Point	21	F	50	FNA	Adenocarcinoma	Suspension
13	Delete	19	F	55	FNA	Adenocarcinoma	Suspension
14	Delete	19	F	43	FNA	Adenocarcinoma	Suspension
15	Delete	19	F	44	FNA	Adenocarcinoma	Suspension
16	Delete	19	M	49	FNA	Adenocarcinoma	Suspension
17	Delete	19	F	49	FNA	Adenocarcinoma	Suspension
18	Delete	19	F	53	FNA	Adenocarcinoma	Suspension
19	Point	21	F	53	FNA	Adenocarcinoma	Suspension
20	Delete	19	F	51	FNA	Adenocarcinoma	Suspension
21	Point	20	F	62	FNA	Adenocarcinoma	Suspension
22	Insert	21	F	62	FNA	Adenocarcinoma	Suspension
23	Point	21	M	51	FNA	Squamous cancer	Suspension
24	Point	21	F	54	FNA	Adenocarcinoma	Suspension
25	Point	21	F	58	FNA	Adenocarcinoma	Suspension
26	Point	21	F	73	FNA	Adenocarcinoma	Suspension
27	Point	21	M	71	FNA	Adenocarcinoma	Suspension
28	Point	21	F	70	FNA	Adenocarcinoma	Suspension
29	Point	21	M	56	FNA	Adenocarcinoma	Suspension
30	Point	21	M	56	FNA	Adenocarcinoma	Suspension
31	Point	21	M	57	FNA	Adenocarcinoma	Suspension
32	Point	21	F	31	FNA	Adenocarcinoma	Suspension
33	Point	21	F	54	FNA	Adenocarcinoma	Suspension
Two mutation site in the same case for No.18 and 19, No.21 and 22. EGFR: epidermial growth factor receptor; FNA: fine needle aspiration; F: female; M: male.							

**2:** *EGFR*、*KRAS*突变情况一览表 *EGFR* and *KRAS* mutation status

	*EGFR* (%)	*KRAS* (%)	Female (%)	Male (%)
Mutation	31 (37.3)	6 (7.2)	24 (60.0)	7 (16.3)
No-mutation	52 (62.7)	77 (92.8)	16 (40.0)	36 (83.7)
Total	83 (100.0)	83 (100.0)	40 (100.0)	43 (100.0)
Mutation rate (%)	37.3	7.2		

检测细胞学标本的同时，有19例福尔马林固定石蜡包埋切片（formalin fixed paraffin-embedded, FFPE）标本作为匹配进行检测。转移淋巴结活检切除标本10例，其结果与细针针吸标本的检测结果一致（kappa=1.0），9例组织学为肺部原发灶标本，细胞学为淋巴结转移灶标本，同一病例原发灶与转移灶两种标本检测结果是一致的（kappa=1.0）。

### 2.3 *KRAS*突变

83例同时进行了KRAS基因检测，基因突变6例，主要发生在2号外显子12和13号密码子上的点突变，突变率7.2%。此6例EGFR均为野生型，未见突变。*KRAS*基因突变检测结果见图 2。KRAS突变情况及突变率见表 2和表 3。细胞学标本检测有基因突变，临床分期为Ⅳ期的13例患者，临床上使用了TKI药物，包括吉非替尼、埃克替尼、厄洛替尼、阿法替尼等。连续用药观察，时间从5个月-36个月，临床疗效为：2例达到完全缓解（complete remission, CR）（16.7%）；8例是部分缓解（partial remission, PR）（66.7%）；3例为最佳稳定疾病（stable disease, SD）（25.0%），SD达到11个月-15个月。从用药情况看，突变位点位于19号和21号外显子的疗效明显，由于例数少，无法计算统计学意义。

**2:**
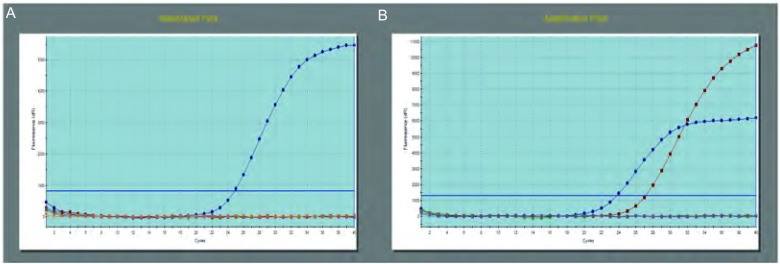
Real-time PCR检测*KRAS*基因突变。A：*KRAS*基因野生型；B：*KRAS*基因突变型。 Real-time PCR detect *KRAS* gene mutation. A: *KRAS* gene wild type; B: *KRAS* gene mutation type.

**3:** *KRAS*的突变情况 *KRAS* mutation status

Mutation No.	Gender	Age	*KRAS* mutation	Sample source	Diag.	DNA source
1	M	68	Point	FNA	Adenocarcinoma	Suspension
2	M	73	Point	FNA	Adenocarcinoma	Suspension
3	M	42	Point	FNA	Adenocarcinoma	Suspension
4	F	53	Point	FNA	Adenocarcinoma	Suspension
5	M	71	Point	FNA	Adenocarcinoma	Suspension
6	F	56	Point	FNA	Adenocarcinoma	Suspension

## 3 讨论

随着分子生物学在临床的应用，基因检测技术的成熟，分子靶向药物在肺癌治疗中的作用越来越受到重视，其疗效也被临床认可。分子靶向药物应用前*EGFR*和*KRAS*基因突变的检测，使用活检切除或者手术后石蜡包埋切片标本的检测结果已经有文献报道^[7-9]^。细胞学浆膜腔积液石蜡包埋块标本，以及16 G-20 G粗针穿刺获取标本制成石蜡包埋块检测*EGFR*和*KRAS*的基因突变在国内也有报道^[10-12]^。以上检测均使用细胞石蜡包埋块标本。当今临床提倡肿瘤患者诊断和治疗应该采取损伤小，患者痛苦少的方法。尤其对于一些就诊时已经是晚期，出现淋巴结转移的患者，临床应用细针吸取获取标本做出诊断后，剩余悬浮液标本能否提取DNA，进行*EGFR*和*KRAS*基因检测，国内尚未见报道。对于晚期淋巴结转移的肺癌患者，细针吸取取材容易，创伤小，是其重要的标本来源。

细胞学标本检测*EGFR*基因突变，突变率为20%-50%^[11, 13, 14]^。均采用灵敏度较高的技术进行检测。何臣等应用酶切富集PCR方法，对30例NSCLC胸腔积液游离核酸*EGFR*基因外显子19缺失和外显子21 L858R突变进行分析，30例NSCLC患者中，*EGFR*基因外显子19缺失10例（33.3%），外显子21 L858R突变5例（16.7%）。丁丽等^[14]^收集23例晚期NSCLC患者恶性胸腔积液，经反复离心后取沉淀细胞行石蜡包埋，巢式PCR法扩增外显子19、20、21，取扩增片段行DNA测序分析。结果显示23例中8例发生*EGFR*基因突变，4例在外显子19，2例在外显子21，2例发生复合突变。7例突变患者服用吉非替尼治疗，中位无进展生存时间达9.5个月。王等^[11]^同样采用密度梯度离心后，将21例胸水细胞包埋，制成蜡块检测*EGFR*基因突变，突变率为42.9%。以上检测病例数均未超过50例，并且采用细胞块包埋方法。本研究是大样本转移淋巴结细针吸取标本检测EGFR和KRAS的报道，采用诊断后剩余悬浮液标本，使用灵敏度比较高的荧光PCR方法进行检测，结果*EGFR*基因突变率为37.3%，*EGFR*基因突变51.5%出现在19号外显子，42.5%出现在21号外显子，与细胞学包埋蜡块标本的结果相一致。

手术切除后组织学标本检测*EGFR*基因突变率为16%-58%^[15, 16]^，本研究突变率在37.3%。研究中19例细胞学针吸同时匹配FFPE切片标本进行了检测，结果显示，转移淋巴结活检切除标本10例，其结果与细胞学针吸标本的检测结果一致（kappa=1.0），说明同一病例不同取材标本的检测结果未存在差异。9例肺部原发灶与淋巴结针吸细胞学标本的检测结果也是一致的（kappa=1.0）。文献报道肺部原发灶和淋巴结转移灶不同部位EGFR检测结果存在差异，本研究这样的病例仅9例，由于例数少，不足以作为统计依据。

突变位于19号和21号外显子，使EGFR受体激酶和对酪氨酸激酶拮抗剂的敏感性增加，癌细胞对吉非替尼的敏感性明显增强^[17]^。细胞学结果显示*EGFR*突变，无病理标本匹配的13例临床分期为Ⅳ期的患者，临床给予TKI药物进行观察，疗效为：2例达到完全缓解CR，此2例基因突变位于19号外显子；8例达到部分缓解PR，5个突变点位于19号外显子，4个位于21号外显子（其中1例有2个突变点）；3例为最佳SD，2个突变位点位于19号外显子，1个位于21号外显子，SD已经达到11个月-15个月。

有关肺癌淋巴结细针吸取标本提取DNA，进行*EGFR*和*KRAS*基因突变的检测，国外已有报道。Billah等^[18]^检测166例NSCLC患者*EGFR*的基因突变，突变率分别是腺癌29.0%，非腺癌7.0%，*KRAS*为23.6%。Lozano等^[19]^检测了138例NSCLC患者的原发灶和转移淋巴结，*EGFR*突变率为17%，*KRAS*为12%，均发现*EGFR*和*KRAS*的突变是相互排斥的。其研究结果反映的是欧美群体。本研究结果83例NSCLC的*EGFR*突变率为37.3%，腺癌细胞为36.7%，4例鳞状细胞癌有2例发生突变，突变率为50.0%，由于鳞状细胞癌例数少，无统计学意义。欧美群体*EGFR*的突变率低于亚洲群体，和手术后组织切片检测结果相一致^[11, 13-17, 20]^。

*EGFR*和*KRAS*基因突变是相互排斥的，文献^[21, 22]^报道*KRAS*基因突变范围为4.7%-13.0%。本研究*KRAS*基因突变率为7.2%，与文献报道相一致，与国内组织学标本的检测结果也是相符合。有报道称KRAS突变率分别为23.6%和12%，明显高于亚洲群体。在肺腺癌中，欧美群体*EGFR*的突变率低于亚洲群体，*KRAS*的基因突变率高于亚洲群体。*EGFR*突变在肺腺癌、女性患者、非吸烟及东亚人群发生率高，吉非替尼在东方人群整体生存率和缓解率明显高于西方人群^[23-25]^。

目前，关于检测*EGFR*、*KRAS*的突变状态作为NSCLC患者TKI疗效的预测指标广为临床所接收，对于不能手术的肺癌患者，如何找到组织标本的替代者逐渐成为探讨热点。本研究结果表明，转移淋巴结的细针吸取可能是较好的组织替代标本，利用诊断后剩余的细胞悬液标本进行检测，标本制作简单、方便，因而具有较高的临床实用性，有助于晚期肺癌患者的个体化治疗。
